# Missed opportunities in TB diagnosis: a TB Process-Based Performance Review tool to evaluate and improve clinical care

**DOI:** 10.1186/1471-2458-11-127

**Published:** 2011-02-22

**Authors:** Nigel Field, Jill Murray, Michelle L Wong, Rob Dowdeswell, Ntomboxolo Dudumayo, Lesego Rametsi, Neil Martinson, Marc Lipman, Judith R Glynn, Pam Sonnenberg

**Affiliations:** 1 Centre for Sexual Health & HIV Research, Research Department of Infection & population Health, University College London, London, UK; 2 National Institute for Occupational Health, National Health Laboratory Service, School of Public Health, University of the Witwatersrand, Johannesburg, South Africa; 3 Division of Pulmonology, Department of Medicine, Chris Hani Baragwanath Hospital, Johannesburg, South Africa; 4 Rustenburg Platinum Mines, Anglo Platinum, Rustenburg, South Africa; 5 Lonmin PLC, Rustenburg, South Africa; 6 Perinatal HIV Research Unit, University of the Witwatersrand, South Africa; 7 Johns Hopkins University Center for TB Research, Baltimore, USA; 8 Respiratory Medicine, Royal Free Hospital, London, UK; 9 Infectious Disease Epidemiology Unit, Department of Epidemiology and Population Health, London School of Hygiene and Tropical Medicine, London, UK

## Abstract

**Background:**

Traditional tuberculosis (TB) treatment outcome measures, such as cure rate, do not provide insight into the underlying reasons for missing clinical targets. We evaluated a TB Process-Based Performance Review (TB-PBPR) tool, developed to identify "missed opportunities" for timely and accurate diagnosis of TB. The tool enables performance assessment at the level of process and quality of care.

**Methods:**

The TB-PBPR tool is a single-page structured flow-sheet that identifies 14 clinical actions (grouped into elicited symptoms, clinical examination and investigations). Medical records from selected deceased patients were reviewed at two South African mine hospitals (A = 56 cases; B = 26 cases), a South African teaching hospital (C = 20 cases) and a UK teaching hospital (D = 13 cases).

**Results:**

In hospital A, where autopsy was routine, TB was missed in life in 52% (23/44) of cases and was wrongly attributed as the cause of death in 16% (18/110). Clinical omissions were identified at each hospital and at every stage of clinical management. For example, recording of chest symptoms was omitted in up to 39% of cases, sputum smear examination in up to 85% and chest radiograph in up to 38% of cases respectively.

**Conclusions:**

This study introduces the TB-PBPR tool as a novel method to review and evaluate clinical performance in TB management. We found that simple clinical actions were omitted in many cases. The tool, in conjunction with a manual describing best practice, is adaptable to a range of settings, is educational and enables detailed feedback within a TB programme. The TB-PBPR tool and manual are both freely available for general use.

## Background

The primary aims of tuberculosis (TB) control programmes are early diagnosis and prompt treatment of infectious cases to limit transmission [1]. To this end, the World Health Organisation (WHO) has developed specific outcome measures to evaluate TB control. Hence, treatment outcomes are recorded internationally and targets of 70% case detection and 85% cure in smear positive pulmonary TB have been set [2]. However, these broad outcome measures do not provide detailed insight into the pathways of clinical care or identify reasons for missing the targets.

Methods of TB diagnosis have not changed significantly for many decades; resting primarily on clinical history, clinical examination, chest radiograph (CXR), and sputum smear and culture. Despite this long experience, there is overwhelming evidence from studies published over the last 50 years that TB diagnosis is prone to significant error [3-9]. Misdiagnosis occurs both if TB is missed and if TB is over-diagnosed. For example, a recent South African study found 21% of adults dying in hospital with a pre-mortem diagnosis of "TB" had no TB at autopsy [10], while in Italy in 1996, 36% of deceased AIDS patients with clinical diagnoses of TB had no evidence of TB at autopsy [11]. On the other hand, and of more concern to public health, are studies from the USA that suggest 5% of notified TB cases are diagnosed only after death [12,13], plus several large autopsy studies showing that TB is missed in life in 18-54% of cases with pathological evidence of active TB [9,14-16]. Avoidable clinical errors can contribute to delays or error in TB diagnosis [17].

This study describes a novel method for evaluating TB control at the point of care using a Process-Based Performance Review tool (TB-PBPR) to identify missed opportunities for early and accurate TB diagnosis. PBPR is a teaching strategy where clinicians retrospectively review patient records to evaluate crucial clinical actions, and has been shown to improve clinical performance [18,19]. Following initial development and piloting in the South African mining industry [20,21], we evaluated the tool by applying it to identify missed opportunities in deceased patients in four different hospital settings.

## Methods

The TB-PBPR tool consists of a single-page structured flow sheet (see additional file 1). Each element is derived from clinical evidence and, as a whole, is in accordance with the 2006 International Standards for TB Care (ISTC) [22]. A manual containing concise, evidence-based clinical summaries was developed for use in conjunction with the TB-PBPR tool to provide guidance on best practice [23].

Recorded data include demographics, clinical and autopsy diagnoses, important clinical actions, missed opportunities and response to therapy. The tool evaluates the integrated process of care for a number of essential clinical actions; first through identification of whether each clinical action was performed, and second through assessment of whether the result of that clinical action was recorded and then acted on appropriately.

In total, the TB-PBPR tool identifies 14 clinical actions which, if carried out, should minimize the number of missed diagnoses: eliciting TB symptoms constitutes 1, clinical examination 6 and clinical investigations 7. "Missed opportunities" are identified as errors causing potential failure to make timely and accurate clinical diagnoses. For example, with regard to CXR, a missed opportunity would be identified if a CXR were omitted, if a CXR were performed but the result not obtained, or if the result were obtained but not acted upon. Where no documentation is found in the clinical notes, the action is recorded as omitted. The tool takes account of the circumstances in which different investigations are indicated because certain investigations may not be required in every patient. For example, lymph node aspiration is not applicable in the absence of lymphadenopathy. When sputum examination identifies TB, further investigations to identify TB are recorded 'not applicable'.

The TB-PBPR tool evaluates a group of 'other' investigations, which should be considered in accordance with ISTC guideline 3 (to investigate extrapulmonary TB), particularly in HIV positive cases with suspected TB and three negative sputum smears [22].

We evaluated the TB-PBPR tool in four hospitals (two South African platinum mine hospitals and two tertiary-care teaching hospitals (one in South Africa and one in the UK)). Cases were selected using different criteria (outlined below) to assess the tool's use in a range of healthcare settings, patient groups and populations. The TB rate is close to 1000 per 100,000 in the general South African population [24], and estimated adult HIV prevalence is 18% [25]. Although TB is less common in the UK than South Africa, the UK-based hospital serves the London community where TB rates (43 per 100,000) are much higher than elsewhere in the country [26]. A medical doctor completed each TB-PBPR flow sheet using the accompanying manual in ~40 minutes.

### Hospital A (South African platinum mine hospital 1)

In this setting, autopsies are conducted for compensation purposes in terms of the Occupational Diseases in Mines and Works Act (ODMWA). Provided next of kin give consent, autopsy is performed in all men dying in employment regardless of the clinical cause of death. Cases not submitted are generally those who die off mine premises. Deceased miners' heart and lungs are removed at their place of employment, placed in formalin and dispatched to the South African National Institute for Occupational Health (NIOH), where histopathology is performed according to a standardized protocol [27,28]. Pathological TB is diagnosed in the presence of necrotizing granulomatous inflammation and/or presence of acid-fast bacilli, other causes having been excluded. Results are stored in the PATHAUT computerised database [29,30].

All patients from hospital A who died and had an autopsy of cardio-respiratory organs at the NIOH between October 2006 and December 2007 (n = 110) were considered for this study. The subset with a clinical and/or autopsy diagnosis of pulmonary TB (n = 62) were selected for review using the TB-PBPR tool. Clinical notes were available for 56 cases.

### Hospital B (South African platinum mine hospital 2)

No autopsies were performed for hospital B. Therefore, all patients who died during 2007 (n = 60) were considered for this study. The subset of those with a natural cause of death were selected for review (n = 35). Clinical notes were available for 26 cases. TB diagnosis was taken from clinical records and made on clinical or microbiological grounds.

A health care service, comprising primary care clinics, specialised clinics and hospital facilities, is provided free of charge to all mine employees at hospitals A and B, approximately 18,000 workers in each case. The healthcare services run their own TB control programmes.

### Hospital C (2700-bed public sector South African tertiary-care teaching hospital)

A convenience sample of 20 deceased individuals with a pre-mortem clinical diagnosis of TB and undergoing autopsy during the period December 2003 to March 2005 at Chris Hani Baragwanath Hospital (CHBH), Soweto as described by Martinson et al [10] was selected for review. Eligibility criteria for the original study included: >18 years of age, survival in hospital for >24 h and next of kin consenting to a full autopsy.

### Hospital D (1000-bed public sector UK tertiary-care teaching hospital)

No autopsy data were available for hospital D. Therefore all patients who were registered with the TB programme at the Royal Free Hospital (RFH), London, and who died between January 2004 and December 2007 (n = 22) were considered for this study. Clinical notes were available for 13 cases, who did not differ significantly in age, sex or ethnicity from cases for whom notes were unavailable.

Ethical approval was obtained from the University of the Witwatersrand. Work in the UK hospital was undertaken as part of a clinical audit of TB services and, therefore, no local ethical approval was required.

## Results

### Patients

We reviewed medical records from 115 patients who died at the four hospitals using the TB-PBPR tool (Table 1). The duration of hospital admission was >24 h in 96% (110/115) of patients. Most patients at the South African hospitals (80-96%) were known to have HIV infection and in the mining hospitals, many had previously been treated for TB. The proportion treated for TB at final admission varied according to hospital setting (57-92%), with treatment started empirically (without microbiological evidence) in 25-53% of cases.

**Table 1:** Characteristics of cases by hospital

	**Hospital A (n = 56)**	**Hospital B (n = 26)**	**Hospital C (n = 20)**	**Hospital D (n = 13)**
**Country, hospital type**	SA, mining	SA, mining	SA, teaching	UK, teaching
**% male**	100	100	48	60
**Age/y; median (range)**	48 (26-64)	43 (20-64)	37 (22-61)	46 (24-69)
**Duration of admission/days; median (range)**	11 (1-110)	7 (1-67)	5 (3-32)	38 (7-85)
**TB treatment completed previously; n (%)**	26 (46)	10 (39)	3 (10)	0 (0)
†**HIV-infected; n (%)**	54 (96)	23 (89)	16 (80)	5 (39)
**TB treated at final admission; n (%)**	32 (57)	16 (62)	14 (70)	12 (92)
**Empirical TB treatment; n (%)**	17 (53)	4 (25)	6 (43)	3 (25)

† HIV status was unknown in 2, 1, 3 and 1 cases in Hospitals A-D respectively

### Clinico-Pathological Comparison

During the study period, 214 deaths were recorded at Hospital A. Autopsy was performed on 110 cases, in whom active TB was found in 40% (44/110). TB was missed in life in 52% (23/44) of cases and was wrongly attributed as the cause of death in 16% (18/110). The sensitivity of clinical diagnosis for TB was 48% (21/44) and specificity 73% (48/66) when measured against autopsy. Data on clinico-pathological comparison for Hospital C cases are published elsewhere [10].

### Symptoms and Clinical Examination

Eliciting of TB symptoms is evaluated by the TB-PBPR tool on the basis of whether 3 symptoms are recorded. Importantly, the tool cannot distinguish between omissions of clinical action and omissions of clinical record. Depending on the hospital, symptoms of chest complaint (cough/haemoptysis/pain) were omitted in up to 39% of patients, weight loss between 15% and 73%, and fever/night sweats in up to 81% (Table 2). The TB-PBPR tool evaluates clinical examination on the basis of 6 clinical actions (Table 2). Chest auscultation was omitted in 32% and 50% of patients from mine hospital A and B respectively, but always performed at both teaching hospitals. Examination for weight loss and lymphadenopathy were performed poorly at all four hospitals, being omitted in 31% to 85% of patients. Notwithstanding clinical omissions, at least one positive clinical finding was documented for most patients: 93% (52/56) at hospital A, 88% (23/26) at hospital B and 100% at both hospital C and D.

**Table 2:** Clinical actions: eliciting of clinical symptoms and examination (%)

	**Hospital A (n = 56)**			**Hospital B (n = 26)**			**Hospital C (n = 20)**			**Hospital D (n = 13)**		
	**Omitted**	**Action performed**		**Omitted**	**Action performed**		**Omitted**	**Action performed**		**Omitted**	**Action performed**	
		**Absent**	**Present**		**Absent**	**Present**		**Absent**	**Present**		**Absent**	**Present**
**Symptoms elicited**												
**Chest complaint**	**38**	**7**	**55**	**39**	**0**	**62**	**15**	**5**	**80**	**0**	**39**	**62**
**Weight loss**	**63**	**5**	**32**	**73**	**0**	**27**	**55**	**0**	**45**	**15**	**8**	**77**
**Fever/night sweats**	**46**	**9**	**44**	**81**	**0**	**19**	**60**	**5**	**35**	**0**	**54**	**46**
**Examination**												
**Chest auscultation** **abnormal**	**32**	**20**	**48**	**50**	**19**	**31**	**0**	**15**	**85**	**0**	**46**	**54**
**Evidence of weight** **loss**	**63**	**0**	**38**	**69**	**0**	**31**	**35**	**0**	**65**	**85**	**0**	**15**
**Pleural effusion**	**39**	**52**	**9**	**96**	**0**	**4**	**80**	**5**	**15**	**39**	**31**	**31**
**Lymphadenopathy**	**86**	**5**	**9**	**89**	**12**	**0**	**40**	**45**	**15**	**31**	**23**	**46**
**Hepatosplenomegaly**	**59**	**27**	**14**	**84**	**15**	**0**	**25**	**35**	**40**	**0**	**31**	**69**
**Neck stiffness/** **confusion**	**71**	**23**	**5**	**73**	**12**	**15**	**45**	**35**	**20**	**15**	**62**	**23**

The TB-PBPR tool was used to evaluate medical records to determine whether clinical actions were omitted or performed. Where an action was performed, we recorded whether symptoms and examination findings were absent or present. For example, in hospital A, clinicians omitted eliciting symptoms of chest complaint (cough/haemoptysis/pain) in 38% of patients. Chest symptoms were elicited in the remaining patients (Action performed); these symptoms were absent in 7% and present in 55% of patients.

### Investigations

Following symptoms and clinical examination, the tool assesses whether clinical investigations were performed appropriately (Table 3). Investigations were omitted in hospitals A to C as follows; CXR in up to 38%; sputum smear in up to 85%; sputum culture in up to 90% (Table 3). However, in hospital D, these investigations were performed in every case. Assessment of other investigations such as lymph node aspiration, pleural tap and lumbar puncture depends upon recording of relevant clinical details. For example, in the absence of examination for lymphadenopathy it was impossible to evaluate the use of lymph node aspiration (marked 'no exam'). Nonetheless, we did identify cases in hospitals A, B and D where lymphadenopathy was present but lymph node biopsy was omitted (Table 3).

**Table 3:** Clinical actions: use of appropriate investigations (%)

	**Hospital A (n = 56)**				**Hospital B (n = 26)**				**Hospital C (n = 20)**				**Hospital D (n = 13)**			
	**NA**	**No exam**	**Omitted**	**Done**	**NA**	**No exam**	**Omitted**	**Done**	**NA**	**No exam**	**Omitted**	**Done**	**NA**	**No exam**	**Omitted**	**Done**
**Chest radiograph**	**0**	-	**38**	**63**	**0**	-	**35**	**65**	**0**	-	**35**	**65**	**0**	-	**0**	**100**
**Sputum Smear**	**0**	-	**52**	**48**	**4**	-	**42**	**54**	**10**	-	**85**	**5**	**15**	-	**0**	**85**
**Sputum Culture**	**0**	-	**75**	**25**	**4**	-	**54**	**42**	**10**	-	**90**	**0**	**15**	-	**0**	**85**
**LN aspiration**	**11**	**80**	**9**	**0**	**23**	**77**	**0**	**0**	**45**	**35**	**15**	**5**	**69**	**8**	**15**	**8**
**Pleural tap**	**66**	**21**	**2**	**11**	**0**	**96**	**0**	**4**	**10**	**70**	**0**	**20**	**69**	**8**	**8**	**15**
**Lumbar puncture**	**54**	**25**	**2**	**20**	**19**	**62**	**0**	**19**	**30**	**40**	**5**	**25**	**46**	**15**	**0**	**39**
**Other**	**25**	-	**68**	**7**	**8**	-	**89**	**4**	**10**	-	**45**	**45**	**54**	-	**0**	**46**

The TB-PBPR tool was used to assess whether appropriate investigations were performed. We recorded 'NA' where the investigation was not applicable (for example, where 'Sputum Smear' had identified acid fast bacilli), we recorded 'no exam' where the relevant clinical examination was not documented, we recorded 'omitted' where an investigation was applicable but not performed, and we recorded 'done' where the investigation was performed. For example, in hospital C, lymph node (LN) aspiration was not applicable in 45% of patients and was not documented in 35% of patients. LN aspiration was omitted in 15% and done in 5% of the patients.

### Missed Opportunities

**Figure 1:**
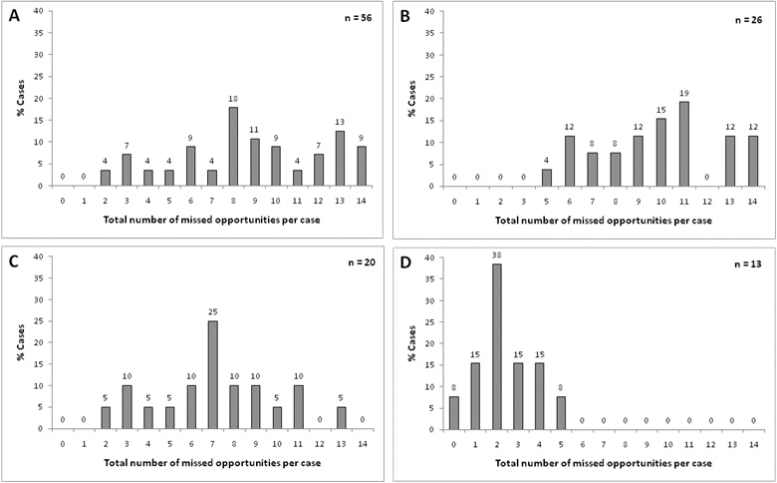
**Histograms showing the distribution of total missed opportunities per case (maximum 14) for each hospital**.

For each of the 14 clinical actions, the tool collapses the chain of clinical process to identify a missed opportunity where the process failed. For eliciting of TB symptoms, the clinical process was considered complete provided one or more symptoms were recorded and appropriate investigations had been performed. We found a mean of 8.8, 9.8, 7.2 and 2.4 missed opportunities per patient at hospitals A-D respectively (Figure 1). For both mine hospitals, we were able to review attendances to the hospital or outpatient clinics in the 3 months before final admission as a surrogate measure of opportunities for earlier intervention. In hospital A, 86% (47/56) of patients had attended at least once while the proportion in hospital B was 92% (24/26). The median number of attendances was 3 and 7 respectively.

## Discussion and Conclusions

We applied a TB-Process Based Performance Review tool as a novel method to evaluate accurate and timely diagnosis of TB. We evaluated the tool in deceased patients, where clinical management may have failed. We therefore expected to find omissions in clinical process, and this may not be representative of the TB programmes in their entirety. Used in conjunction with autopsy results, as for hospitals A and C, the tool deconstructs a patient's clinical care to capture diagnostic errors. In the absence of autopsy data, as for hospitals B and D, the tool still provides valuable insight into patient care and overall TB programme performance. Where documentation was missing, we recorded an omission because clear documentation is essential for clinical governance as well as communication reasons. We found that simple but fundamental diagnostic clinical actions such as chest auscultation, CXR and sputum smear were not recorded in many cases. The tool highlights specific areas for improvement within each setting. Similar system failures have been reported in South African urban state clinics [31]. In the absence of a systematic clinical history, examination, and appropriate investigation, the evidence base for logical diagnostic decision-making is lost. This is important in TB (particularly with HIV co-infection) where cases can present diagnostic challenges even where all appropriate investigations seem to have been explored [8]. Similarly, implementation of a 19-point checklist of simple clinical actions was associated with significant improvements in surgical outcome [32].

Many of these 115 patients presented with symptoms that should have prompted clinicians to consider TB (Table 2); overall 62% presented with chest symptoms, 38% with symptoms of weight loss and 37% with fever or night sweats. Other indications of the possibility of TB existed in these patients, 34% had previously been treated for TB and 85% were HIV-infected. All admitted patients survived for at least 24 h (many survived for longer). Clinicians therefore had opportunity to initiate investigations and treatment, and in many cases to monitor the response to treatment. Furthermore, in hospitals A and B, we found most cases made contact with medical services in the three months before final admission. We would expect to find further missed opportunities in these earlier patient/clinician interactions.

We highlight that sputum culture results were missing for so many of the South African patients, although this investigation was available in all hospitals. The dangers of multidrug resistance have been well described [33], and a high index of suspicion must be maintained, particularly where patients fail to improve on TB treatment or have recurrent TB (ISTC, standard 14 [22]). On the other hand, HIV testing was performed well, acknowledging the importance of ascertaining HIV status where TB is suspected.

Different criteria were used for selection at each hospital, with the important distinction that (in this study) patients at Hospitals C and D were diagnosed with TB pre-mortem. However, a differential diagnosis that included TB was common to all. Although the selection criteria varied, and this limits comparison, the four hospitals do allow some useful broad observations to be made. We demonstrate that the tool identifies missed opportunities in a variety of settings, both in public and private hospitals, and in developed and developing countries. We recognise that not all missed opportunities carry equal weight but found the total number of missed opportunities to be a useful assessment of overall performance. The findings in hospital D, although the number of patients was small, suggest that it may be possible to achieve low numbers of missed opportunities for some patient groups, and this supports previous data showing that, despite low rates of error, some deaths are unavoidable [17]. Omissions in clinical care occur throughout the world and are not limited to TB; one study found that 45% of US adults do not receive care that is consistent with current recommendations [34]. If simple clinical actions are omitted in these settings, where TB prevalence is high, this raises concerns about settings where clinicians have far less experience of treating TB.

The TB-PBPR tool was designed and used here for in-patients and may require adaptation before use in some low income outpatient settings where clinical records may be less detailed. However, many of the key missed opportunities such as basic history taking and sputum smear are common to nearly all settings. Further evaluation will assess whether the TB-PBPR tool can be used to improve local clinical and programme management.

Successful TB control requires basic clinical and public health management to be performed efficiently and consistently. Clinical omissions and misdiagnoses have implications for both the individual and the community, delaying treatment and increasing the period of infectivity leading to increased transmission, treatment failure, medical costs, and deaths.

The tool was designed with an educational objective in mind, to be used by clinicians to reflect on clinical practice and monitor missed opportunities. The TB-PBPR tool may be particularly useful to improve clinical care in patients with a range of poor outcomes, (e.g, development of drug resistance and recurrence) and its use is not limited to deceased patients. We suggest that the tool may augment broader WHO measures for TB programmes because it allows detailed evaluation to feedback into a TB programme and improve clinical care.

## Competing interests

The authors declare that they have no competing interests.

## Authors' contributions

JM, PS and NF designed the study. NF, JM, MW and ND completed the TB-PBPR tool flow sheets, and the data were collated by NF. All other authors participated in the design and coordination of the study. All authors contributed to the manuscript, read and approved the final manuscript.

## Pre-publication history

The pre-publication history for this paper can be accessed here:

http://www.biomedcentral.com/1471-2458/11/127/prepub

## Supplementary Material

Additional file 1 **The TB-PBPR tool**. Click here for file
