# Supplementary material for: Missed opportunities in TB diagnosis: a TB Process-Based Performance Review tool to evaluate and improve clinical care
Source: BMC Public Health. 2011 Feb 22;11:127. doi: 10.1186/1471-2458-11-127 (PMC3051909; doi:10.1186/1471-2458-11-127)
Supplement: Additional file 1 — The TB-PBPR tool. [file 1471-2458-11-127-S1.DOC]

**Supplementary Figure.**TB-PBPR Tool
